# Advantages, Pitfalls, and Developments of All Optical Interrogation Strategies of Microcircuits *in vivo*

**DOI:** 10.3389/fnins.2022.859803

**Published:** 2022-06-28

**Authors:** Stylianos Papaioannou, Paolo Medini

**Affiliations:** Physiology Section, Department of Integrative Medical Biology (IMB), Umeå University, Umeå, Sweden

**Keywords:** network functional imaging, holographic optogenetics, all-optical circuit interrogation, multiphoton microscopy, *in vivo* electrophysiology

## Abstract

The holy grail for every neurophysiologist is to conclude a causal relationship between an elementary behaviour and the function of a specific brain area or circuit. Our effort to map elementary behaviours to specific brain loci and to further manipulate neural activity while observing the alterations in behaviour is in essence the goal for neuroscientists. Recent advancements in the area of experimental brain imaging in the form of longer wavelength near infrared (NIR) pulsed lasers with the development of highly efficient optogenetic actuators and reporters of neural activity, has endowed us with unprecedented resolution in spatiotemporal precision both in imaging neural activity as well as manipulating it with multiphoton microscopy. This readily available toolbox has introduced a so called all-optical physiology and interrogation of circuits and has opened new horizons when it comes to precisely, fast and non-invasively map and manipulate anatomically, molecularly or functionally identified mesoscopic brain circuits. The purpose of this review is to describe the advantages and possible pitfalls of all-optical approaches in system neuroscience, where by all-optical we mean use of multiphoton microscopy to image the functional response of neuron(s) in the network so to attain flexible choice of the cells to be also optogenetically photostimulated by holography, in absence of electrophysiology. Spatio-temporal constraints will be compared toward the classical reference of electrophysiology methods. When appropriate, in relation to current limitations of current optical approaches, we will make reference to latest works aimed to overcome these limitations, in order to highlight the most recent developments. We will also provide examples of types of experiments uniquely approachable all-optically. Finally, although mechanically non-invasive, all-optical electrophysiology exhibits potential off-target effects which can ambiguate and complicate the interpretation of the results. In summary, this review is an effort to exemplify how an all-optical experiment can be designed, conducted and interpreted from the point of view of the integrative neurophysiologist.

## Purpose and Content of the Review

Many neurophysiologists went or are going through the methodological passage “from the electrode to the photon,” as nicely and effectively summarized and predicted by Scanziani and Häusser 13 years ago (Scanziani and Hausser, 2009). As we will briefly highlight in this review the vast majority of the key concepts in neurophysiology have been discovered by means of electrophysiological techniques, which remains the “golden standard” as essentially neurons are electrically excitable cells that communicate between each other via electrical signals. However, it became quite quickly evident that electrical signals produce (or are produced by) other phenomena that can be measured optically (we will recapitulate the main milestones of this development as well). The development of optical techniques has reached a similar or in some case even higher degree of cell type specificity, with comparable spatial, and, in some cases, temporal resolutions. Recent development of multiphoton network imaging combined with holographic optogenetics allows activity measurements and manipulation of functionally identified neurons in the microcircuit in order to interrogate the function of the circuit components identified either functionally, genetically or based on their input/output connectivity, *without the use of any electrode*. Here, we consider *“all optical” approaches those based purely on multiphoton microscopy to perform functional imaging and manipulation, in absence of any electrophysiological measurements*. The scope of the review is to go through the main milestones of this approaches, describe more recent developments *in relation to their physiological relevance for system neuroscience laboratories* and questions, implying with that, that we will also discuss how those development could realistically be available for the community on a larger scale. In doing this, we will go through the current and developing constraints of “imaging only” and “photostimulation only” approaches, as the two components are essential parts of what is strictly defined as “all optical.” Special attention will be then given to advantages and issues related to the *combination* of the two approaches, as of strict definition of the “all-optical” investigations. Single photon microscopy is not considered as the focus of the review because it is multiphoton microscopy – due to its “intrinsic confocal character”- that allows *flexible, function-based* single-cell resolution within the subnetwork to be imaged and/or manipulated. For example, all-optical approaches leveraging on a combination of multiphoton functional imaging or electrophysiology and single-photon optogenetics for closed-loop optical approaches [such as (Newman et al., 2015; Prsa et al., 2017), respectively] will not be discussed in the context of this review. However, for the above mentioned reasons, electrophysiology will often be used as reference comparison throughout the text, when such a comparison is meaningful and relevant.

## Historical Milestones Toward All Optical Physiology

### The Development of Modern Electrophysiology as “Golden Standard” for Physiologists

The fundamental role of electrical signals in physiology was already contemplated by Isaac Newton, and the experimental proof of the electric nature of muscle contraction and signal propagation in nerves is due to Luigi Galvani (1786). Since then, the progress in developing electrophysiological methods goes hand-in-hand with the most important breakthroughs in our understanding of brain function. The development of technology to amplify and record from single neural fibres led to the discovery of sensory evoked neuronal activity by Adrian and Sherrington (Sherrington’s 1906 monograph and Nobel Lectures). Later, the achievement of intracellular recordings by Hodgkin and Huxley lead to the conception of one of the most successful model in Neuroscience describing generation and propagation of action potentials (Schwiening, 2012). During the 1960’s with the help of extracellular recordings from anesthetized cats, Hubel and Wiesel discovered the functional organisation of the primary visual cortex (V1) and provided a mechanistic explanation on how tuning properties of V1 neurons emerge (Hubel and Wiesel, 1959, 1962). The development of chronic electrode arrays endowed us with the ability to record population neural activity from behaving animals. This approach provided the first mechanistic explanation of higher cognitive functions, when the work of O’Keefe (O’Keefe and Dostrovsky, 1971) and of Moser (Hafting et al., 2005) led to the discovery of place and grid cells by observing the activity of a population of neurons in hippocampus and entorhinal cortex, respectively, during navigation in freely moving rats. Although endowed with more limited sampling capacity, intracellular recordings *in vivo* allowed a better understanding of the principles by which subthreshold synaptic inputs received by principal neurons are converted to given spike outputs in the living brain (e.g., Margrie et al., 2002; Steriade, 2006), also in behaviourally relevant contexts (e.g., Houweling and Brecht, 2008). The advancement of electrophysiology assisted by the progress in electronics after WWII offered a wide toolbox of methods to the contemporary investigator. These methods cover the whole range from patch clamp to investigate the intracellular electrical properties and integrative function of cells and subcellular structures such as dendrites and axons to the use of miniaturized probes with several thousands of recording sites revealing the firing patterns of ∼10.000 neurons, spanning several cortical and subcortical areas, simultaneously during freely behaving animals (Steinmetz et al., 2019). Despite the impressive advancement of the electrophysiology, it suffers from several inherent drawbacks. It is an invasive approach since it requires physical proximity between the recording probe and the neural tissue. Despite the excellent spatial and unprecedented temporal resolution, electrophysiology techniques do not scale as easily when it comes to the size of the brain area that can simultaneously be sampled. Moreover since the probes that need to be inserted or implanted have different moduli of elasticity from the brain tissue, they are not optimal for moving animals due to the relevant motion between the probe and the brain which by itself further amplifies the mechanical invasiveness of the approach. Even though recent advancements minimize invasiveness by rendering electrodes ultrathin (Forni et al., 2021), by introducing feedback-driven piezo system to compensate and minimize the damage caused by brain pulsations (Fee, 2000), or by shaping electrodes so to assume a juxtasomal configuration (Spira and Hai, 2013; Shmoel et al., 2016), the problem of mechanical invasiveness remains.

### Imaging Brain Activity: From Intrinsic and Voltage Dye Imaging to Calcium Network Multiphoton Microscopy

Optical approaches to understand brain function are by definition mechanically less or not invasive, unless requiring aspiration of brain structure or prism/lens insertion in the parenchyma to reach deep brain structures (e.g., Andermann et al., 2013). Apart from their use in anatomy, optical methods have been utilized to study neural tissue function. Already in the late 1940s Hill and Keynes reported changes in light absorption from the surface of a stimulated nerve (Cohen et al., 1970) that allowed to reconstruct the time course of an action potential (Koike-Tani et al., 2020). This “label free” approach has never been performed successfully *in vivo* up to now, but is also relevant to a larger scale as diffusion tensor MRI, originate in part from similar activity-dependent cellular swelling mechanisms (Koike-Tani et al., 2020). Yet another optical approach to infer function from imaging of label-free tissue is achieved with the help of optical signals related to light absorbance changes associated with metabolic and hemodynamic processes. These “intrinsic signals” that follow neuronal activity confirmed the cortical columnar organisation (Bonhoeffer and Grinvald, 1993; Malach et al., 1993), and are also responsible for the BOLD signal in fMRI (Rector et al., 2009). However, these signals lack cellular resolution and display a very slow activation dynamics (e.g., ca 10–20 times slower compared to electrical activity activation dynamics (Medini, 2011)).

Intrinsic signals exhibit a very low signal-to-noise ratio (SNR) which is further worsened under *in vivo* or awake conditions due to further biological noise (from heart and ventilation-related pulsations, animal movements, etc.). In an effort to enhance the yield of optical imaging of voltage fluctuations, pioneers such as Cohen, Grinvald and Loew developed fluorescent voltage sensitive dyes that intercalate in the phospholipid bilayer of the neuronal membrane and emit fluorescence in proportionality to the transmembrane voltage, exploiting the fact that although the transmembrane voltage changes are small (e.g., 100 mV during one action potential), the electrical field is high as the membrane is very thin [see (Braubach et al., 2015) for an historical review, and for some last relevant developments with the more recent “blue” dyes introduced by Amiram Grinvald see (Shoham et al., 1999; Grinvald et al., 2016)]. After staining the brain tissue with such dyes and using a fast, sensitive and low noise camera or photodiode arrays one can reliably record changes in fluorescence that represent the underlying voltage, including subthreshold, fluctuations (Grinvald et al., 2016). This approach measures largely subthreshold (synaptic) inputs as the largest amount of molecules are in the neuronal compartment with the largest area/volume ratio, the dendrites (Petersen et al., 2003; Newton et al., 2021). As we will highlight below, the field is now characterized, by very interesting developments in genetically encoded voltage indicators [GEVIs, from the first indicator introduced by Siegel and Isacoff by fusing a GFP in a voltage-dependent channel – up to the last, more effective variants – reviewed by Bando et al. (2019b) and Knopfel and Song (2019)].

Calcium activity is of great interest in neuroscience mostly because its dynamics corresponds to suprathreshold activity (Kerr et al., 2005; Siegle et al., 2021), due to the fact that it is one of the ion species showing highest ratio between baseline and activity-driven concentrations while having the lowest resting membrane permeability (Oh et al., 2019) and also due to the superlative role in processes like plasticity. Calcium imaging became a relevant and useful tool to study the physiology of electrically excitable cells after Tsien and colleagues introduced in 1985 (Grynkiewicz et al., 1985) the ratiometric calcium indicator Fura-2, showing that upon calcium binding the Fura 2 molecule has a change in the excitation peaks at 340 and 380 nm, the ratio being proportional to internal calcium change. Moreover, the existence of an isosbestic point (that is a wavelength at which the emission is not calcium sensitive) made measurements independent of concentration as well as from factors affecting the absolute amount of fluorophore, such as cell thickness, etc. For *in vivo* measurements, the non-ratiometric calcium indicator OGB-1 became largely used due to the large signal-to-noise ratio. Moreover, the acetomethoxy (AM) ester form of calcium dyes could easily cross cell membranes and bind with the cytoplasmic calcium, where it is cleaved to active, calcium-sensitive form. This development simplified the intracellular staining of neurons which could be realized *in vivo* by a simple injection of an organic calcium dye such as OGB in the area of interest [“bolus loading” -(Stosiek et al., 2003; Ohki et al., 2006)], whose fluorescence was shown to reliably report neuron’s spiking activity *in vivo* (Kerr et al., 2005). The success and mass adoption of calcium network imaging, apart from the development of AM ester dyes was due to another breakthrough that was developing in parallel: in 1990, Denk, Strickler and Webb published their pioneering work on two-photon absorption microscopy (Denk et al., 1990). This approach, which was based on the theoretical work of Maria Göppert in 1930, pushed the boundaries of microscopy to another level. Two-photon absorption provided unprecedented spatial resolution and tissue penetration with reduced phototoxicity essentially due to the fact that two-photon excitation is limited to focal point and that radiation of lower energy, longer wavelength penetrates the brain more in depth due to less scattering (Svoboda and Yasuda, 2006). The combination of intracellular staining with AM-esters of calcium dyes with two-photon network imaging *in vivo* provided for the first time ever a sufficiently fast, single cell resolved, imaging of a local population of neurons in the intact brain of anesthetized and awake, head fixed, behaving animals. This was arguably one of the most important achievements that lead to the introduction of all-optical network imaging in systems neuroscience, which is in place, in a refined form, even today. The introduction of genetically encoded calcium indicators (GECIs), again by Roger Tsien, offered the possibility of longitudinal observations of the very same networks over time with the sensor being expressed either virally or in transgenic animals. Cameleon was the prototype of the fluorescence resonance energy transfer (FRET)-based GECIs, where two chromatically distinct GFPs were linked by a calmodulin so that calcium binding allowed FRET to happen between the two fluorophores (Miyawaki et al., 1997). Other FRET-based sensors were later introduced and tested *in vivo*, such as Tn-XLL by Oliver Griesbeck (Mank et al., 2008). The most diffused GECIs nowadays used belong to the other family of “single-fluorophore GECIs” like GCaMPs. In these, a circularly permuted GFP in the middle links two domains that are kept distant from one another: upon calcium entry the two side domains interact tightly and displace water molecules from GFP rendering it fluorescent. New variants with different affinities, on and off kinetics have been created up to version 8 (Zhang, 2018). The recent introduction of red-shifted GECIs (Dana et al., 2016, 2018), allowed to reach the infragranular, main output cortical layers, mostly due to reduced scattering of red-shifted radiations both in excitation and emission on one side, and allowed performing artefact free all optical interrogation of cortical circuits in combination with blue-shifted opsins (Forli et al., 2021).

Thus, the development of genetically encoded reporters of neural activity [calcium (Chen et al., 2013), voltage (Bando et al., 2019a)] with fluorescent proteins optimized for excitation with NIR irradiation have significantly increased the fidelity, dynamic range and SNR allowing us a reliable optical read-out of neuronal activity. In combination with the multitude options to drive the expression of these reporters based on different properties such as cell type, laminar distribution, activity level, anatomical connections, etc., this approach has provided the contemporary neurophysiologist with a vast armament to design and conduct highly tailored experiments for the question at hand.

### Optogenetics and Holographic Photostimulation

Focal stimulation of the brain has always been a tool to investigate the hypothesis of the anatomical placement of functionally defined modules, starting from the stimulation experiments of Penfield. Intracortical microstimulation techniques allow a more focal stimulation of the functional network and has been shown to bias perceptual decision [see for ex. the seminal work of Salzman in the primate parietal areas involved in motion perception (Salzman et al., 1992)]. Further studies documenting the sparse level of activity in the cortex (in particular in supragranular layers) then showed that rodent can actually perceive the juxtasomal activation of a single neuron (Houweling and Brecht, 2008). Of relevance, recent all optical physiology experiments showed that holographic photoactivation of a few neurons can evoke behaviourally detectable perception (Dalgleish et al., 2020). The possibility to stimulate neurons with light became evident when Deisseroth and Boyden (Boyden et al., 2005) showed that this was possible by transfecting microbial opsins in neurons, a work that opened the field of *optogenetics*. Also, the possibility to photoinhibit neurons rendered for the first time in neuroscience possible a bidirectional, cell type-specific control of neuronal activity, as testified by the introduction of the chloride pump Halorhodopsin (Zhang et al., 2007), of the proton pump Archaerhodopsin, as well as by powerful chloride-permeant opsins [reviewed in Deubner et al. (2019)]. Of note, channels mimic also the shunting effect of GABA (increase of membrane conductance), compared to ionic pumps [that instead inhibit only via hyperpolarisation), and thus have been proven to be generally more effective (Wiegert et al., 2017)]. Optogenetic stimulation can also generate artificial percepts (e.g., Histed and Maunsell, 2014) and showed that specific cell types have a causal role in simple behaviours (e.g., Lee et al., 2012). However, single-photon, wide field optogenetics lacks cellular resolution: in particular functionally identified neurons by their response properties cannot be targeted.

In this respect a fundamental work to move toward single cell optogenetics *in vivo* showed the possibility to stimulate opsins via multiphoton absorption (Rickgauer and Tank, 2009). SLMs (spatial light modulators) are programmable diffractive devices inserted in the microscope path that alter the beam wavefront to spatially redistribute the focused beam in beamlets that target multiple neurons in 3D. Computer generated holograms are used to display the desired phase mask on the SLM, using usually the iterative Fourier based Gerchberg-Saxton algorithm (Gerchberg and Saxton, 1972), although new algorithms are now being proposed to improve calculation velocity and to optimize intensity distribution patterns (Hossein Eybposh et al., 2020; Jin et al., 2021). Opsin activation can happen by simultaneous (e.g., spiral) scanning of spots (Packer et al., 2015) or via a scanless approach (Papagiakoumou et al., 2010). Although the group of Yuste showed that, within the same preparation and hence with the same opsin (in that case C1V1), a scanless approach requires in general two-fold more power (Yang W. et al., 2018), it is also true that the same group reports that these differences tend to disappear with short illumination times. It is thus conceivable to think that in the case of very quick, not integrating opsins, the use of scanning vs. scanless technology might bring about comparable results, although many experimental variables have to be taken into account such that the computer generated hologram used, as well as how prone is the specific algorithm to speckle [reviewed in Ronzitti et al. (2018)]. Initially scanless photoactivation was combined with raster scanning for imaging (Dal Maschio et al., 2010) and often temporally integrative, red-shifted opsin such as C1V1 were used in combination with GCAMP6 imaging (Packer et al., 2015; Yang W. et al., 2018). Later opsins with faster and more reliable temporal kinetics were used (Ronzitti et al., 2017; Shemesh et al., 2017). More recent works have combined the use of the highly sensitive ChroME (Mardinly et al., 2018) and ChRmine (Marshel et al., 2019) with calcium imaging to extend the number of photoactivated cells, up to the very recent demonstration of the capacity of control of many hundreds of neurons with high spatio-temporal fidelity also with a scanless approach using the latest ChroME2.0 variants (Sridharan et al., 2022). Finally, it is also possible to combine all optical photo-inhibition and holographic stimulation *in vivo* at high efficiency (Forli et al., 2018) (see also Mardinly et al., 2018). The expectation in the following years is that closed-loop approaches [such as in Zhang et al. (2018)] will unravel new organizing principles behind cortical computations.

## Spatio-Temporal Resolution, Constraints and Developments of All-Optical Physiology

### Temporal Resolution and Fidelity of All-Optical Techniques

Achieving sufficient temporal resolution while recording or imaging neuronal activity is important in order to be able to produce results that are biologically relevant to the underlying question. For example in questions underlying neuronal coding and more specifically *temporal coding*, we often demand millisecond time resolution. On the other hand, when the question at hand concerns the relevant amplitude of population responses between two areas during a task, then we can afford time resolution in the range of hundreds of milliseconds or even seconds. In electrophysiological approaches, temporal resolution is limited by the amplifier’s temporal filters, which in turn is dependent on the amplification gain but also on the acquisition rate of the analog-to-digital converter. In any case, sub-millisecond resolution is easily achieved. In imaging on the other hand, which is an indirect approach, temporal resolution is limited by the scanning rate but also the temporal properties of the activity indicator (calcium or voltage in most cases).

Imaging scanning rates can vary from < 1 up to 10 Hz, depending on the scanned area, for galvanometric scanning and up to 30–60 Hz for the resonant one or when the scope has an electrically tunable lens (Grewe et al., 2011). Relatively new techniques utilizing Acousto-Optic Deflectors can scan in a 3-dimensional random access mode with rates up to several kHz (Duemani Reddy et al., 2008; Grewe et al., 2010; Nadella et al., 2016; Szalay et al., 2016). In most cases, it is the mechanical inertia of the scanning mirrors that limits the rate of imaging. A promising remedy to this limitation is the utilisation of scanless imaging where the imaging beam is moved with the help of a Spatial Light Modulator which splits the beam in multiple beamlets and calculates and implements the appropriate interference pattern in order to move the beam and scan the area of interest (Nikolenko et al., 2008; Dal Maschio et al., 2010). Moreover the development of technological demonstration of custom-made systems that enables us to scan with kHz resolution is of great interest and provide a preliminary view of possible future developments to come (Kazemipour et al., 2019; Tsyboulski et al., 2019). Very relevant at this regard is also the strategy proposed by Na Ji, where an array of spatially distinct and temporally delayed optical foci is formed in a way to allows line scans at laser repetition rate (MHz), via a module that could be *potentially* added to commercial scopes (Wu et al., 2020). Based on these scanning rates, the theoretical upper bound for the reconstruction of the inferred spike activity from the calcium data ranges from few (0.5–5) Hz up to KHz for the fastest approaches like the ones mentioned above.

As mentioned above, calcium imaging is an indirect method and as such the temporal characteristics of the calcium indicator also contribute to the theoretically maximum temporal resolution. The temporal kernel of the gcamp6 resembles a non-linear, exponential function with a fast, few millisecond rising phase tau and a slow, in the range of hundreds of millisecond decaying phase tau (Chen et al., 2013). The above implies that in order to infer spikes from calcium transients we need to first deconvolve the summated calcium transients and then infer the spike activity from the deconvolved, temporally diluted calcium events.

The slow decay tau further degrades temporal resolution but also provides an advantage in the accuracy of inferring spike activity imaged with slow frame rates. This is true because the GCaMP6 convolves the fast spikes with the slow off kinetics, thus leaving a slow temporal trace of the fast events. For example, in the theoretical case where 5 spikes occur within 50 msec during a 10 Hz imaging, they could all be missed since the period of scanning, 100 msec, is longer that the duration of the 5 spike event, 50 msec. The slow temporal tail of the GCaMP6 thought, allow us to pick up the decaying phase of the calcium transient and from there deconvolve and infer the spiking activity. As a conclusion, the disadvantage of the slow temporal characteristics of GCaMP6 provides the opportunity to summate and filter in time fast events so they can be acquired even with slow imaging frame rates.

A new era is currently under development with the availability of genetically encoded voltage indicators (GEVI) (Bando et al., 2019a; Knopfel and Song, 2019; Villette et al., 2019; Li et al., 2020). Although promising, GEVIs are not yet as mature as the calcium indicators which have undergone significant improvements and are currently in their current 8th generation (Murat et al., 2021). Also in order to utilize the temporal bandwidth and resolution that they offer in a higher number of labs, scanning technology to routinely achieve sub-millisecond resolution should become more widespread. Yet another weakness of the Gevi’s is their need for significantly more powerful laser on the sample as well as the lower signal to noise ratio. With regard to that, the paper from Villette et al. (2019), employing the GEVI ASAP-3, represents a significant advancement, as signals of 5–10% are reported under 20 mW illumination. However, in order to overcome the still present photobleaching problem and the limited displacement of molecules the authors used a non-conventional scanning system, as they applied non-stationary frequencies to an AOD system to scan a holographic pattern of excitation spots in 3D so to expand the excitation volume. Undoubtedly, in the near future further molecular “maturation” of GEVIs and the more widespread use of adequate scanning technologies (see examples above) will provide the new standard for all optical neurophysiology with sub-millisecond resolution, and subthreshold responsiveness comparable to the current electrophysiology standards. With this respect, recent optical developments –potentially applicable to standard microscopes- allows to scan at kHz rates and perform GEVI imaging *in vivo* (Wu et al., 2020).

### Spatial Extent and Resolution of All-Optical Interventions

Electrophysiological approaches allow for a wide range of spatial extent of the recorded area. Wideband extracellular signals can be filtered accordingly to our interest, for example low pass signals, i.e., local field potentials (LFP) reveal a weighted sum of pre- and postsynaptic activity from a source that can originate mms away from the recording site and integrates the synaptic activity of a cortical area of several hundred μm in diameter (Haider et al., 2016). Previous work (Okun et al., 2010) shows that the propagation distance and integration area of LFP signals depend on the dominant frequency in a way that lower frequencies propagate further and also integrate signals from larger areas. On the other extreme, selecting the signal with the highest temporal frequency, we limit the source only to the adjacent to recording site cortical tissue allowing us to pick up signals from a single or few cells. This property of extracellular electric signals allows us to vary the spatial extent of signal integration by simply choosing the appropriate frequency pass-band. The downside of this is that when choosing lower frequency and spatially integrated signals, the acquired signal corresponds to the weighted sum of all the spatial sources. That means that there is a significant compromise to the spatial resolution by collapsing the sources of a several hundreds of μm area to a single point.

#### “Field of View”

Electrophysiological approaches require physical proximity between the structure to be recorded and the electric probe. This mere fact limits the extent of scalability of the electrical approach *per se*: Inserting more probes within a given cortical tissue volume inevitably increases the mechanical invasiveness- trauma as well as the biological reaction as expressed by increased number of glia, known as gliosis. This in its turn leads to gradual degradation of the recording quality. The above limitation is not present in the optical approaches in which the imaging beam is scanned, by different means, on the cortical area to be imaged. Due to this advantage, imaging endows us with an unsurpassed potential for mechanically non-invasive, large spatial extent, i.e., field of view. On this effort several labs have developed custom made microscopes with up to 25 mm^2^ field of view (Sofroniew et al., 2016; Yu et al., 2021) enabling the near simultaneous recording of single-cell resolved activity from multiple visual areas in the mouse neocortex. This tool is vital for probing inter-areal interaction and acquiring large scale data which is a necessary step toward understanding the dynamics of the cortex as a whole (see concluding remarks on causality inference). The current limitation of this approach at the moment is the need of designing custom objective lenses to compensate the aberrations introduced by the needed wide scan angles. A larger field of view is also important for holographic photostimulation. The field of view for photostimulation is determined by the SLM angle of diffraction. Modern SLMs have more pixels and permit larger diffraction angles and hence larger field of views, so to cover several mm^2^ of cortical surface [see for example the custom-made SLM in Marshel et al. (2019), where also multiple SLMs were used to multiplex different holograms in different layers], and also larger pixel size so to minimize interpixel interference and hence ensuring a higher diffraction efficiency.

#### Depth

When it comes to reach of depth, electrical recordings can be acquired from any structure as long as it can be accessed by a probe. The problem of increasing mechanical damage, as well as other issues mentioned above are still in place so in most cases, when looking for deep structures we use single shanks with multiple recordings sites. This approach brings into our reach subcortical structures like thalamus and striatum (Pachitariu et al., 2016) but requires *post hoc* evaluation of the recording area.

Two photon microscopy utilizes NIR laser which is scattered and absorbed less in the brain tissue compared to visible light so it offers a significantly extended reach of depth compared to single photon imaging. Despite that, in practice, the deepest cortical layers are still beyond our reach. So there is a great demand for deeper imaging. We thus review some of the recent advances that can contribute to an extended reach of depth. Functional imaging in the main infragranular, deep cortical layer has been initially achieved by prism implantation (e.g., Andermann et al., 2013), and subcortical structures have been successfully imaged by GRIN lenses (Qin et al., 2020). These remain, however, mechanically invasive approaches. Interestingly, to reach optically deeply located structures, molecular efforts have been done to develop extremely sensitive opsins, as testified by the possibility to photostimulate midbrain and brainstem structures (up to 7 mm depth) expressing ChRmine even via transcranial stimulation (although with powers of 400 mW/mm^2^) (Chen et al., 2021). Performing 3D holography and imaging *in vivo* will be challenging in these conditions largely due to scattering-related hologram degradation issues, to be addressed in the future.

One way to tackle the excitation laser power decay due to tissue scattering and absorption is the use of regenerative amplifiers. These amplifiers generate laser pulses with lower frequency, several kHz instead of 80 MHz but higher peak powers. This configuration allows us to keep the average power within the accepted range (10–50 mW), thus minimizing thermal side effects, while increasing the peak pulse power and increasing the efficiency of two-photon excitation (Mittmann et al., 2011). In other words, there are way fewer but significantly more powerful pulses of laser that can tolerate better power degradation due to tissue scattering and absorption. Functional network imaging in layer 5/6 has been performed but at the expense of temporal resolution because pulsing the laser in the kHz imposes an upper limit to scanning. Overcoming scattering imposed by the increasing optical inhomogeneities during deep imaging is also important to increase the efficiency of two-photon excitation. This has been shown by using either organic (Tischbirek et al., 2017) or genetically encoded (Zhao et al., 2011; Akerboom et al., 2013) calcium sensors with red shifted excitation and emission spectra (thus reducing the scattering in both phases). Another way around has been the use of adaptive optics with an approach similar to the one used in astronomy: in advance deforming the wave front of the laser beam so to counteract the phase aberrations introduced by the tissue (Wang et al., 2015). Recent advantages in femtosecond lasers have led to the production of sources with extended wavelength, up to 1,700 nm. The availability of high peak energy, low repetition rate, extended NIR pulses provided the ability for three-photon excitation of calcium indicators such as GGaMP6 (e.g., Yildirim et al., 2019; Wang et al., 2020). Three-photon imaging achieves unprecedented tissue penetration depth which brings into our reach structures below 1 mm from pial surface [reviewed in Ouzounov et al. (2017)]. A possible problem to be anticipated is the possibility of even larger spectral overlap of the three-photon absorption spectra of sensors and actuators for all optical approaches, a problem that will have to be addressed in the future.

Is the future of deep imaging continuing to increase peak powers in order to continue to image deeper? Such an approach has certainly an upper limit in the necessity to reduce pulse frequencies and hence the acquisition speed. Hopefully future approaches will also focus on increasing the collection efficiency instead, as the current estimates of the percentage collection of the emitted photons indicate there is significant (three-fold) margin for improvements (Zinter and Levene, 2011).

In all optical physiology, in some cases, it can be physiologically relevant to photostimulate a high number of cells, in particular when aiming to replicate spontaneous patterns on a mesoscale level [although there are clear evidences that the number of neurons necessary to elicit a perception is in the order of a dozen in the visual cortex (Dalgleish et al., 2020), of 20 in the olfactory bulb (Gill et al., 2020), on top of observations indicating the existence of possibly supralinear inhibitory mechanisms limiting the activated/activable area (Rolotti et al., 2022)]. This can be achieved by using lasers with higher peak powers (and lower repetition rate to maintain the average power within biologically safe limits). The reduced repetition rate increases the photostimulation efficiency by using “integrating” opsins with a longer tau off such as C1V1. The second approach is to split the beam in a higher number of beamlets, which implies delivering more power to the sample. The use of highly sensitive opsins, characterized by large single channel conductance and short tau off (Mardinly et al., 2018; Marshel et al., 2019), can reduce the power demand and ameliorate the concerns on tissue heating (see next paragraph). On the other side, it is also important to highlight that stimulating a very high percentage of neurons simultaneously is not physiological neither might be needed (Marshel et al., 2019) - see also (Dalgleish et al., 2020).

#### Resolution

Achieving sufficient spatial resolution both in electrical and optical measurements is important if we aim to attribute the measured activity to identified cells and cell types or subcellular compartments like dendrites. Extracellular electrophysiological approaches allow for single cell resolution, as for example with cell-attached recordings and single unit extracellular recordings. In order to achieve sub-cellular resolution, the need of intracellular access is needed. With the help of patch clamp techniques, apart from somata, we can record activity from dendrites (e.g., Smith et al., 2013) and axons (Hu and Shu, 2012), i.e., we can achieve sub-cellular resolution. A significant drawback of the electrophysiological techniques is that they are intrinsically blind. This limits our ability to visually target cells to record from, unless we augment the electrophysiological approach with microscopy-assisted visualisation of the positions of the putative targets and probe. So, the importance of imaging in achieving sub-cellular resolution or the ability to select the target to record from is paramount.

Imaging approaches achieved a great leap in improving spatial resolution with the introduction of two-photon microscopy in which the lateral resolution is diffraction limited. The spatially restricted, within few μm, point spread function (SPF) of the excitation beam made possible the targeting and resolving of single cells or even cell compartments such as dendrites/spines. Possible issues of signal contamination that degrade the spatial resolution can originate from the axial resolution which is inferior to the lateral one due to spatial distribution of probability of multiphoton absorption caused by the optical properties of the objective lens, compared to the lateral, and can lead to simultaneous excitation of parts of axially adjacent somata or other cell compartments. Several post-processing methods have been developed to resolve this issue such as neuropil subtraction (Keemink et al., 2018; Kuznetsova et al., 2021). Yet another advantage of two-photon microscopy is the option to create tailored-made shape of psf in order to increase the yield and the rate of imaging. For example, extending the z-dimension of a psf can help imaging dendrites which are not always co-planar with the imaging beam (Szalay et al., 2016). The above can be achieved with the help of Bessel-like beams or spatial light modulators (Theriault et al., 2014; Lu et al., 2020). Increasing spatial resolution is important also for the specificity of holographic stimulation, as neighbouring cells can have different functional response properties [“salt and pepper” spatial microarchitecture -e.g., rodent primary (Mrsic-Flogel et al., 2007) and association (Olcese et al., 2013) visual cortices]. Molecular strategies like including a somatic restriction tag in the construct (such as a motif in the amino acid sequence of the Kv2.1 channels) helps to restrict the expression to somata and primary dendrites (thus reducing the photostimulation of neighbouring dendrites, also belonging to other neurons, not to mention axons on passage). As high levels of expression can anyhow cause contamination to distal dendrites, new strategies have also been developed (Shemesh et al., 2017) [surprisingly, localizing opsins at the axonal initial segment did not show promising results (Grubb and Burrone, 2010)]. Other strategies have been used to target opsins to axons or to dendritic compartments (see below). A higher spatial resolution of holographic stimulation can be achieved by spatially confinement of the holographic photostimulation -particularly critical along the *Z*-axis. This has been achieved either by spiral scanning multiple somatas (e.g., Zhang et al., 2018) or by introducing temporal focusing [reviewed in Papagiakoumou et al. (2020)].

## Physiologically Relevant Pro and Cons of All-Optical Approaches in System Neuroscience, Compared to Corresponding Electrophysiological Approaches

### Pros of “All Optical”

*-* All optical approaches allow first of all to employ *a broader variety of physiologically meaningful readouts*, that is beyond calcium sensors, it is nowadays possible to measure directly the transmembrane voltage with the help of GEVIs (Bando et al., 2019a; Knopfel and Song, 2019), or the concentration of several relevant second messengers [such cAMP (Harada et al., 2017)] which are essential for many cell functions. Recently, sensors able to detect the release of specific neuromodulators such as dopamine (Patriarchi et al., 2018) have become available. With this regard, it is of great interest the work done by the group of Yulong Li on developing new G-protein-activation coupled optical sensors to reveal the release of neuromodulators crucial for cognitive, emotional and sleep regulation *in vivo* under multiphoton microscopy such as dopamine (Sun et al., 2020) or serotonin (Wan et al., 2021). Importantly, this series of sensors have subcellular resolution, subsecond kinetics and respond in physiological ranges of concentration, in relevant areas in behavioural contexts/challenges known to evoke such releases. The concentration of physiologically relevant cations other than calcium can be measured {as in the case of sodium (Ona-Jodar et al., 2017) [see also (Naumann et al., 2018) for more recent indicators] or potassium (Sui et al., 2015), and the same holds true for chloride (Sulis Sato et al., 2017)]. One critical parameter to check for any new sensor is whether the linear range of sensitivity of the indicator falls within physiological variations that is to compare the indicator affinity with the physiological range of concentrations of the molecule of interest. This has been indeed a relevant parameter in the case of development of genetically encoded chloride sensors such as the Chlomeleons, which has been reported to have a dissociation constant of about 160 mM at physiological pH (Kuner and Augustine, 2000), and thus creating problem in the imaging chloride transients at the low concentrations expected in adult neurons. Superchlomeleon has certainly represented an advancement with this regards (Kd in the order of 20–40 mM) (Wimmer et al., 2015), and recently accurate measurement of *in vivo* concentrations of both Chloride and pH have been performed in the developing cortex with the new ChlopHensor (Sulis Sato et al., 2017). Additionally, it is now possible to measure *in vivo* glutamate release optically thanks to the design of functional glutamate sensors usable under a multiphoton microscopy (Borghuis, 2019). In general, as we mentioned, it is important to estimate that the linear range of these sensors falls within the physiological concentrations. As we will later discuss for other sensors, future work will be on the other side needed to deep our knowledge on up to which degree the presence of such reporters, that also function as endogenous *buffers*, could interfere with the normal cell and network physiology in relevant behavioural contexts.

- The *level of attainable cell type specificity is also higher* in the case of all optical techniques, as they rely on cell type specific conditional expression techniques (see also for further references Emiliani et al., 2015). Combinatorial molecular approaches, relying on single-or double-conditional constructs, eventually coupled with transynaptic strategies and/or activity-dependent reporters allow to specifically label cells based on their function, connectivity and genetic properties up to the possibility to restrict labelling to those neuron subpopulations functioning as Boolean logic operators [AND, OR, IF; see as reference example (Fenno et al., 2014)]. Note that also electrophysiology allows to target single cell recordings based on tagged opsins (Optopatcher technique), but with an experimental yield and variation possibilities not comparable with all optical techniques [reviewed in Katz et al. (2019)].

- The *mechanical invasiveness is lower* in the case of all optical approaches, although a craniotomy and possible meningeal inflammations can be a serious concern and limit the experimental yield also in the case of all-optical interventions. Needless to say, the mechanical invasiveness directly on the brain parenchyma is higher in the case of electrophysiological electrode arrays. It is necessary to highlight that light, beyond certain power thresholds, exerts side effects on the brain parenchyma via a complex variety of mechanisms. Some of these effects impact on the capacity of the sensor to be excited (bleaching) and increase up to certain extent in parallel with phototoxicity, partially due to shared mechanisms (Tauer, 2002) (see below for the relevant paragraph).

- The fourth advantage of all-optical approaches is the *higher spatial resolution*, that is the capacity to measure the activity in identified subcellular compartments (e.g., dendrites Jia et al., 2011; Smith et al., 2013; Wilson et al., 2016; Kerlin et al., 2019, somata, axons Jaepel et al., 2017; Leinweber et al., 2017). The holographic intervention has a comparable spatial resolution, attainable due to high spatial resolution of modern SLMs combined with new molecular techniques aimed at restricting expression at somata, dendrites or axons [reviewed in Rost et al. (2017)]. Thanks to these advancements, both levels of input integration (network, dendrite) can be investigated optically nowadays.

### Cons of “All Optical”

- The *temporal fidelity of calcium imaging is still 1–2 orders of magnitude lower* compared to conventional electrophysiological measurements. As we highlighted above, this is not necessarily problematic when the neuronal phenomena under investigation can be conveniently described by measuring firing rates at population level, but in those physiological situations – or sensory systems- in which spike timing can instead contain critical information (e.g., Panzeri et al., 2001; Wehr and Zador, 2003; Higley and Contreras, 2006), or when sparse firing levels can be relevant (Dalgleish et al., 2020), the use of electrophysiology is still preferable.

- The second limitation of calcium imaging (at somatic level, in the context of functional network imaging, is that it is *not adequate to report the subthreshold activity at single neuron level during network imaging*. Although the process of synaptic integration has been studied in several contexts *in vivo* by means of dendritic calcium imaging (see examples above), this can be done for isolated neurons, and should be done always taking into account the consideration that there are physiological differences in the spatio-temporal profiles of voltage and calcium activation along the very same dendrite (Roome and Kuhn, 2018). Most significantly, even though the neuropil can provide an EEG-like signal, somatic calcium signals reflect largely the suprathreshold activity (Kerr et al., 2005).

As a perspective, the very much needed molecular advancements in GEVIs have the potential to solve both issues. When coupled with adequately fast scanning and recording systems [such as AODs (Duemani Reddy et al., 2008; Grewe et al., 2010; Nadella et al., 2016; Szalay et al., 2016), scanless-SLM-imaging (Nikolenko et al., 2008; Dal Maschio et al., 2010) or temporal multiplexing for which a feasibility study is now available (Tsyboulski et al., 2019)], the availability of GEVIs with a higher quantum yield allowing higher signal-to-noise ratios would overcome both problems. Indeed, as mentioned what is critically missing are sufficiently sensitive GEVIs (indeed, there are technical solutions to accelerate the acquisition so to match it with the reporter’s temporal fidelity). However, recent improvements on this front are certainly promising (Adam et al., 2019; Piatkevich et al., 2019; Villette et al., 2019; Li et al., 2020).

- The third limitation of all optical technique is that it relies on expressing exogenous constructs in neurons. Thought this is attained by means of non-replicative AAVs, t*he expression of calcium chelators and exogenous opsins can have pathological consequences*. Expressing proteins in the cell membrane (actuators) or in the cytoplasm (calcium reporters) that are not originally present in the cell, does have several functional implications. It has been reported that chronic expression of Gcamp6 causes aberrant electrical activity in terms of epileptiform spikes in several transgenic lines (Steinmetz et al., 2017). Such abnormalities might be due to Cre or tTA-related toxicity (Schmidt-Supprian and Rajewsky, 2007), but to larger extent the main cause is the cytoplasmatic accumulation of the calcium sensor interfering with calcium homeostasis [e.g., the calmoduline module of GCAMP6 interferes with gating of L-type Ca2+ channels (Yang Y. et al., 2018)]. Moreover, abnormal axonal morphology and targeting of ChR2 expressing cells has been documented, as well as altered excitability (Miyashita et al., 2013).

- Finally, one has to take into account *some degree of interference between the expression and/or function of optosensors and optoactuators* as shown by the fact that the response to photostimulation correlates inversely with the level of expression of calcium sensor (Packer et al., 2015; Supplementary Figure 6A).

- The interaction described above is at the level of the expression, and should be distinguished from *the “crosstalk” problem due to the fact that the imaging laser can also photoactivate the opsins* if the two-photon excitation spectra are not sufficiently separated and/or if the power on spot is too high (Packer et al., 2015; **Supplementary Figure 2**). Traditional ways of minimizing the problem is to limit the power of the imaging line (which might limit to some extent the SNR), or scanning over a large field of view so to reduce the dwell time on each soma. Such crosstalk can be overcome by combining the use of “spectral orthogonality” between the sensor and the actuator, i.e., aiming for a larger degree of spectral separation compared to the “original” pair C1V1 – GCAMP6, as recently proven by successful efforts to increase the efficiency of all optical approaches (Forli et al., 2018, 2021) (e.g., using blue-shifted opsins for photostimulation and red-shifted calcium sensors for functional network imaging). Also relevant with this regard, more favourable wavelengths to minimize the crosstalk between the “classical couple” GCaMP6 and the C1 V1 have been recently reported. The possibility to further extend the palette of sensors and actuators is exemplified by works like the recent one characterizing variants of the fast, red-shifted opsin ChRmine that allowed to perform 3-colour all optical physiology (at one-photon though) (Kishi et al., 2022). In relation to fact that in the future three-photon microscopy will be probably more used to perform optical measurements in deep brain structure, the problem of the spectral cross talk will possibly be worsen as the three-photon excitation spectra of fluorophores can be broader than the ones measured at two-photon (e.g., Liu et al., 2020).

- The *spatial resolution of all optical stimulation techniques has become better* [e.g., by soma-targeting-strategies, that still might have some leakage at least in the primary dendrites; or by introducing temporal focusing in scanless approaches – which can reach a spatial confinement similar to galvo-approaches -reviewed in Adesnik and Abdeladim (2021)], *but there is still margin for improvement.* However, the problem remains and can be minimized by reducing the opsin level of expression, by using a sparse expression strategy (if experimentally acceptable), by using opsins with shorter decay time (that is, less integrating over time) (Chen et al., 2019), or more sensitive opsins which require less power/cell. Reducing the power/neuron is important as the volume (especially the Z axis extension) where two-photon excitation occurs (that is the spatial resolution, being the two photon microscope without pinhole) increases with excitation power, although this phenomenon is less pronounced with high numerical aperture objectives (see Rubart, 2004, simulation of Figure 2).

### Experiments Possible Only With “All Optical Approaches”

The “Pros” of all optical approaches can be at best demonstrated by briefly discussing those type of experiments that would otherwise be impossible with other approaches. Among these we can mention different recent types of experimental works tackling previously not addressable, fundamental question in neuroscience, and for each category we will provide examples of new knowledge that otherwise couldn’t have emerged.

(a) Experiments in which *the activity of functionally identified neurons is manipulated/replicated to test whether a behavioural function associated with the area investigated is causally linked to identified subcircuits*. This type of approach is necessary in rodents, including mice, as they lack a columnar organisation in V1, which still represent a model system to study functioning and plasticity of cortical microcircuits. Indeed, rodent (visual) cortex, instead of having a defined columnar structure, exhibits a “salt and pepper” distribution of functional response properties (e.g., Ohki et al., 2006; Mrsic-Flogel et al., 2007; Olcese et al., 2013), rendering necessary to use 3D optogenetic holography to opto-modulate the desired functional subcircuits.

In the case of the cerebral cortex, work from the group of Häusser showed that the number of cells to be optically stimulated to trigger behavioural detection is rather limited (in the order of dozen) (Dalgleish et al., 2020), and that the impact of photostimulation on a visual detection task on properly tuned neurons depend on the visual task difficulty [the easier the task the less facilitating was photostimulation -(Russell et al., 2019)]. The group of Yuste investigated the role of the specific cortical ensembles in mouse V1 activated by visual stimuli during a visual discrimination task. They had a special focus on the neurons with pattern completion properties, that is on those neurons capable of triggering the whole ensemble. Interestingly, and again in favour on the weight of sparse coding in neocortex, they found that photoactivation of a pair of such neurons effectively activated the whole ensemble and brought about to the corresponding, correct behaviour (Carrillo-Reid et al., 2019). Still in line with the idea that activation of a few cells can bias considerably the local network, Chettih and Harvey measured how neighbouring neurons reshaped their stimulus representation (influence mapping) upon single neuron photostimulation, finding often evidence of suppressive interactions that were more pronounced in the case of tuning similarity (Chettih and Harvey, 2019). Recent work on the mouse (pre)motor cortex has also shed new light on the “persistent firing” mechanisms behind working memory in the prefrontal cortex, another intriguing question in cognitive neuroscience since many years (Daie et al., 2021). Also, recent work finally clarified the precise function and mechanism of action of the inhibitory cells in layer 1 in sensory processing (Fan et al., 2020).

The fundamental questions in neuroscience are often similar in different sensory systems: a classical one is the i*dentification of the parameters of the neuronal activation in the network that actually code for stimulus properties*. A recent work in the olfactory bulb identified that synchronicity of activation is more important as determinant to trigger sensory perception compared to latency of stimulus presentation in relation to breathing, and showed that also in this sensory system the activation of ca 20 neurons is enough to drive behaviourally detectable perception (Gill et al., 2020).

Another neuronal circuit in which holographic efforts have been done is the hippocampus, and in particular the efforts have focused on the demonstration of causality of place cells and spatial memory. Robinson et al. (2020) used an all optical approach to manipulate functionally identified hippocampal place cells that encoded behaviourally relevant locations in a VR environment. Targeted stimulation of few neurons was enough to bias spatial memories, providing a fundamental causal link between place cells and spatial memory. Rolotti et al. (2022) recently addressed the same question, but with an interesting angle on the mechanisms behind the plastic capacity of the place cell network. CA1 pyramid are indeed rapidly plastic and can form new place field within single trials. They holographically induced quickly forming place fields during spatial navigation, with induction efficiency being correlated with the spatial density of activated neurons and inversely related to inhibitory network recruitment.

(b) Yet another promising field of all optical interrogation is the *study of dendritic integration in vivo*. Two photon microscopy already allowed to study the role of dendritic mechanism in enhancing the receptive properties of neurons, or conveying the behavioural saliency of sensory input in behaving animals (e.g., Kerlin et al., 2019). The recent application of multiphoton voltage sensitive dye imaging at dendritic level and its coupling with optogenetic holography from the lab of Yuste is of extreme interest in this regard (Cornejo et al., 2022). Scanning modalities optimized for the convoluted dendritic structure *in vivo* have been designed and implemented (e.g., Gobel and Helmchen, 2007, also via use of specific AOD approaches Szalay et al., 2016; Villette et al., 2019,- see also Wu et al., 2020).

(c) *Unraveling the physiological weight of transynaptically labelled connectivity in defining functional response properties of identified neurons*. As we have mentioned above all-optical techniques allow also to read and manipulate the activity not only of functionally identified neurons, but also of neurons characterized based on their specific connectivity. An example of such work is presented by the recent study of Geiller et al. (2022), where AOD imaging was done in the input cells to study their receptive field plasticity during navigation and place cell plasticity. Combining transynaptic technologies with all-optical approaches will allow in the future the emergence of new knowledge on the functional connectivity of functionally identified brain microcircuits [see also (Wertz et al., 2015) with an AOD approach].

## The Relatively Unexplored Issues of Phototoxicity, Photobleaching and Photointerference in All Optical Physiology

As written above, all optical interrogation techniques are for sure mechanically less invasive, but there are some specific concerns related to cellular health related with (over)expression of exogenous constructs, which seem particularly pronounced with virally mediated expression (see above paragraph). Another relevant dimension in this discussion is the one of interaction between light – and in particular near-infrared radiation (NIR) – and the brain parenchyma.

### Photobleaching and Phototoxicity

The reversible process of excitation and fluorescence emission of a fluorophore can be interrupted by the irreversible generation of an excited non-fluorescent variant of the original fluorophore, which is in turn able to generate reactive oxygen species. This is one of the main mechanistic links between photobleaching and phototoxicity. Indeed, reactive oxygen species can cause for example lipid peroxidation which often irreversibly alters cell membrane permeability (Tauer, 2002). Importantly, phototoxicity can happen also independently of bleaching, due to the capacity of light of adequate wavelengths to directly interact with physiologically relevant biomolecules [e.g., UV-driven thymine dimerisation of DNA, a process relevant with three photon microscopy with 780 nm due to the UV emission (Tauer, 2002)]. Although photobleaching (and toxicity) are negligible in optical planes above and below the focal one in multiphoton compared to confocal microscopy, due to the fact that excitation is virtually limited to the focal plane, the “local photodamage” is for exactly the same reason significant in the vicinity of the focal plane. Indeed, whereas excitation increases with the square of the power, often photobleaching increases with the cube of the power in case of multiphoton microscopy (Patterson and Piston, 2000), suggesting a further degree of non-linearity. The high peak powers used in three-photon microscopy could generate formation of plasma (high numbers of free electrons) with local vacuolisations, white luminescence and eventual carbonisation (Tauer, 2002). Other works suggest that cell photodamage in multiphoton microscopy scales with P/t^2^, where P is the average power and t is the pulse duration (Tauer, 2002). It is easy to grasp that the process of phototoxicity is often the integral of different components which normally have different mechanisms and hence different temporal dynamics. The quickest changes are photochemical induced changes (including the ones coming from photobleaching), direct interference of light with neuronal molecules and ionic channels, and the immediate results of heat-induced damage and of focal photocoagulation. Other cytopathological processes are slower as they happen as consequence of lipid peroxidation destabilizing both the internal and external membranes and are thus related to massive inflow of sodium and calcium (calcium enters also via NMDA-activation dependent on pathological local hyperactivity). Massively increased calcium concentrations can induce either necrotic or apoptotic cell death. Recent work tried to quantify phototoxicity at this level (by labelling and quantifying the expression of protease-caspase-activation, heat shock protein activation, glial activation markers): the result was that for this type of extreme, cytopathological phototoxicity (characterized by apoptosis induction!) the threshold is about 250 mW of continuous illumination for ca 15 min (Podgorski and Ranganathan, 2016).

### “Photointerference”

Beyond these (almost) irreversible forms of phototoxicity, it is important to stress that there is ample but still controversial literature on possibly more reversible, “modulatory” effects of NIR, often (but not necessarily) mediated via heat. It has been estimated that illumination of the brain surface with 100 mW causes ca 1.8 celsius degree of heating which reaches a steady state within a few minutes (Podgorski and Ranganathan, 2016). Noticeably this increase is variable depending on the spatio-temporal pattern of photostimulation (Picot et al., 2018). This power is “only” two-fold below compared to the ones at which pro-apoptotic cascades are activated, and is at the higher margin of the power used in network imaging [wide field holography can reach several hundreds of mW (Yang W. et al., 2018)]. These levels of NIR must have biologically relevant consequences if one takes into account that even 10 mW scanning can induces calcium transients in astrocytic processes (Schmidt and Oheim, 2020), a process capable to induce glioexocytosis of powerful neuromodulators (e.g., Lalo et al., 2014).

These large scale, network effects of NIR-radiation seem to be largely induced by thermal radiation and not by photoabsorption. It is important to highlight that there are also more direct effects of NIR on nerve cell activity. For example, NIR is shown to block action potential conduction along axons (Walsh et al., 2016), in cultured neurons (Feyen et al., 2016) and that this is coupled to blockade of potassium voltage-gated channels, although a direct modulation of sodium channels has also been shown (Li et al., 2014). Noticeably, the field remains controversial as there are clear experimental indication that NIR can depolarize and induce spike in artificial membranes (Shapiro et al., 2017), can activate ganglion neurons (Paris et al., 2017), can induce calcium transients in neurons (Kaszas et al., 2021), or increases visual cortical responsiveness in primates as assessed by intrinsic signal imaging and extracellular electrophysiology (Cayce et al., 2014). Other reports show instead a decrease in visual responsiveness in rat visual cortex (Wu et al., 2013), as well as decreases of intrinsic signal response in rat somatosensory cortex (Cayce et al., 2011). As far as visible light is concerned (used in single photon optogenetics and imaging), the sources and the illumination conditions often used in optogenetic experiments can induce potassium-dependent hyperpolarisations whose consequences are behaviourally detectable (Owen et al., 2019), whereas other report shows light-dependent activation (Stujenske et al., 2015). Older reports indicate that visible light, at intensity levels comparable to those expected inside the rodent brain, can induce release of the inhibitory neurotransmitter GABA from slice preparations (Wade et al., 1988). Last, but not least neurons in the brain expresses endogenous photoreceptors which are similar structurally and functionally to retinal rhodopsin [encephalopsins (Blackshaw and Snyder, 1999)]. Such non-visual opsins play a role in circadian rhythms and regulates physiologically relevant metabolic processes such lipolysis in relation to hibernation (Guido et al., 2022). Having said this, a possible neuromodulatory role of non-visual opsins has yet to be shown, neither it is clear whether non-visual opsins absorb NIR at the power present to the skull). Overall, the *net* effect of all the above processes on neuronal and network activity has to be yet properly documented in the living brain in standardized conditions, close to the ones used in all optical physiology.

## Concluding Remarks and Desirable Future Developments of Physiological Relevance

### All Optical Approaches and Causality Inference in System Neuroscience

The final aim of cell-type specific optical interrogation of cortical microcircuits is to be able to draw causal, mechanistic links between the function of identified microcircuits (that is composed by different cell types) and simple (to start with) behaviour forms, admitting that the distinction between simple and complex behaviour necessarily makes sense. Manipulating activity is nowadays accessible to many labs and is extensively used. As shown by recent all optical works, knowledge is already contributing to improve our understanding on the principles of cortical functioning in relation to behaviour. However, in drawing causal links, a few principles have to be taken into account:

- *The brain is a highly complex, adaptive system and any type of manipulation reconfigures and homeostatically re-sets the system to the new state*. This means that the effects of one manipulation can bring the brain in a different state (e.g., implying different attentional or perceptual thresholds), making difficult to unambiguously interpret the disappearance of one behaviour or the appearance of a new one as causally driven by the manipulated microcircuit (Otchy et al., 2015). A trivial example could be given by the fact that the alteration of electrical activity in one epileptic cortical temporal association region at the beginning of a seizure causes a person to stop writing: this would not allow to draw the conclusion that the cortical region is causally involved in writing. A more economical explanation is that the brain is in a new status which makes it stop doing an ongoing activity. As an example, the group of Ölveczky (Otchy et al., 2015) showed that acute inactivation of one sensory-motor nucleus in birds disrupts vocal behaviour not *per se*, but due to silencing of another premotor nucleus upstream. However, chronic lesioning to the same sensory-motor nucleus did not cause behavioural problems due to compensatory homeostatic plasticity.

- The second dimension of the problem is that *the biological reality is that we intervene on is made up by many, concatenated loops, and this is particularly true for cortical circuits, where almost every interarea connectivity has the reciprocal, feedback one*. Simply put, we intervene on and we try to explain a loop and not a unidirectional interaction. This makes conclusion safer in case of unidirectional, straightforward connections (from ex to certain subcortical structures, e.g. cortico-striatal connections).

- The third dimension is that *brain circuits display plasticity and redundancy*, (that is neighbouring circuits taking over, being facilitated in this by having a partially redundant function), which also can complicate the effects of both acute and chronic manipulations.

It has been argued (Wolff and Olveczky, 2018) that it is only by knowing the interareal connectivity, and *by comparing the effects of acute vs. chronic lesions that causal conclusions can be carefully drawn*. Moreover, conclusions are safer in case of simple straight-forward unidirectional connectivity, this is the case for peripheral and subcortical structures.

Indeed, to draw correct causal connections, it is necessary to take into account the effects of the manipulation of the directly affected neurons onto the neighbouring ones in the same microcircuit, as well as to the effects observed in the target region (on a mesoscopic scale). This is why multiple-area, or wide area scopes (the so called *mesoscopes) can be so important to draw causal conclusions*, in particular in the cortical physiology field (see paragraph on temporal resolution). A better understanding of the “optical interference” of light on neuronal activity (see paragraph 5) will also help to interpret results.

It is very important to highlight that all-optical techniques offer the unique opportunity to *replicate physiological patterns of activity in neuronal ensembles*, a manipulation that definitely can to some extent minimize the concerns expressed above, as the off target effects are probably more pronounced when an acute, sudden, simultaneous silencing or activation of one structure is made (as it happens with massive, one-photon optogenetic stimulation). This also highlights a pro of photostimulation strategies compared to photoinhibitory ones (we cannot know when a spike would have come *in vivo*).

### Future Developments of Physiological Relevance

Häusser and Scanziani were right in predicting a bit more than a decade ago that the major tool of system neuroscience would have gradually moved from the electron to the photon (Scanziani and Hausser, 2009). From the above discussion, testifying the tremendous efforts and achievements of all optical physiology *in vivo*, we would like to summarize in form of summary list what we think could represent field advancements that would be either necessary methodologically to enhance the “physiological power” of the all optical approaches or other developments that would facilitate and extend the adoption of all optical technologies in a significantly higher number of system neuroscientists’ labs:

• *diffusion of mesoscopes (with field of view of ca 5 mm) and adaptation of 3D holography to it*; would allow to monitor various interconnected areas, so to better interpret the causality link between the initial optostimulation and the final behavioural change. This will imply the implementation of multiple SLMs or new technical development allowing that (Yang et al., 2015; Marshel et al., 2019);
• still at the light of minimizing off-target effects, holography has the capacity to mimic natural pattern of activation, so to possibly minimize side, “off-target” effects. To *further increase temporal fidelity of stimulation, digital micromirrors* devices could be used: they can be driven significantly faster than current SLMs (but they have less efficiency) (Bhatia et al., 2021);
• exploiting and testing *more chromatically distinct combinations of optical sensors and actuators so to minimize cross talk* problems during all optical physiology, in line with the interesting results of the Fellin lab (Forli et al., 2018, 2021);
• extending the use of *transgenic lines of tested health and characterized by stable expression in order to reduce AAV-related hyperexpression/toxicity problems*, *or invest on molecular strategies to overcome such slow toxicity processes*. Needless to say, moving forward in that direction would represent a fundamental need for any possible, thinkable clinical trial;
• develop even *more effective GEVIs* (with higher quantum yield and reduced sensitivity to photobleaching), and, in parallel;
• extend and also making efforts to commercially develop the use of *adequately fast scanning techniques to commercial microscopes* [e.g., AOD-systems or Na Ji’s FACED module (Wu et al., 2020)];
• establish protocols to measure the efficacy of photoactivation to targeted levels while monitoring off-target effects, with a capacity to “optically clamp” the network at desired levels. At this respect the *implementation in commercial microscopes of closed loop approaches such as the one designed by the Häusser lab* would be of tremendous importance;
• deepen our understanding of the nature and *effects of acute and chronic effects of light and heat on brain networks in vivo* (including at the above described level of “*photointerference*”). *This knowledge will also be a necessary prerequisite for any potential clinical trial with optogenetics;*
• in relation to this latter point *developing technological solutions, also implementable in commercial scopes, to increase the photon collection efficiency* rather than continue to increase the (peak) powers used from the source lasers;
• investigate the *feasibility of all optical physiology at three photon*, its upper spatio-temporal resolution limits, as well as its possible spectral windows at the light of currently available sensors/lasers, as well as its phototoxicity for each specific sensor/actuator protocol;
• further develop *multi-photon fiberscopes of miniaturized multi-photon microscopes* (Helmchen et al., 2001; Sawinski et al., 2009; Zong et al., 2021), and testing how holography could work via fibers [for more developed discussion on this perspective see also (Adesnik and Abdeladim, 2021)]. This step would be obviously fundamental to extend all optical approaches to more ethologically relevant behavioural tasks.

## Author Contributions

Both authors listed have made a substantial, direct, and intellectual contribution to the work, and approved it for publication.

## Conflict of Interest

The authors declare that the research was conducted in the absence of any commercial or financial relationships that could be construed as a potential conflict of interest.

## Publisher’s Note

All claims expressed in this article are solely those of the authors and do not necessarily represent those of their affiliated organizations, or those of the publisher, the editors and the reviewers. Any product that may be evaluated in this article, or claim that may be made by its manufacturer, is not guaranteed or endorsed by the publisher.

## Abbreviations

AAV: adeno-associated virus
AM-ester: acetoxymethyl-ester
AOD: acousto-optic deflector
ATP: adenosine-triphosphate
BOLD: blood oxygen level-dependent
cAMP: cyclic adenosine monophosphate
ChR2: Channelrhodopsin 2
DNA: deoxyribonucleic acid
EEG: electroencephalogram
fMRI: functional magnetic resonance imaging
FRET: fluorescence resonance energy transfer
GABA: Gamma-aminobutyric acid
GECI: genetically encoded calcium indicator
GEVI: genetically encoded voltage indicator
GFP: green fluorescent protein
GRIN: gradient index
LFP: local field potential
MRI: magnetic resonance imaging
NIR: near-infrared radiation
NMDA: N-methyl -D-aspartate
OGB-1: Oregon green BAPTA-1
PSF: point spread function
SLM: spatial light modulator
SNR: signal to noise ratio
UV: ultraviolet
V1: primary visual cortex.
